# Parenting Self-Efficacy in Immigrant Families—A Systematic Review

**DOI:** 10.3389/fpsyg.2020.00985

**Published:** 2020-05-26

**Authors:** Joanna Boruszak-Kiziukiewicz, Grażyna Kmita

**Affiliations:** Department of Clinical Psychology of Child and Family, Faculty of Psychology, University of Warsaw, Warsaw, Poland

**Keywords:** parenting, self-efficacy, immigrant families, acculturation, migration

## Abstract

**Introduction:** Parenting self-efficacy (PSE) refers to parents' belief in their ability to perform the parenting role successfully, and derives from Bandura's concept of personal self-efficacy formulated within the social cognitive theory. PSE has been demonstrated to be a strong predictor of parenting functioning. At the same time, relatively less is known about its possible role in the situation of migration, when a family experiences acculturation stress in the process of adaptation to the new culture. Therefore, the aim of this systematic review was to summarize available data on the conceptualization, measurement, and the role of parenting self-efficacy in the context of acculturation processes, and in various groups of immigrant parents.

**Methods:** An extensive search of eight electronic databases was conducted in August 2018 and updated in February 2020 to identify peer-reviewed articles on parenting self -efficacy among immigrants. Eleven studies met pre-specified criteria for inclusion. Nine of the studies employed a quantitative design, whereas the remaining two studies used qualitative methods. In three of the quantitative studies, interventions/programs for immigrants were assessed.

**Results:** Three different approaches to conceptualizing and measuring PSE were identified in the analyzed papers: domain-general, domain-specific, and narrow domain. Incongruent results were found with regards to the links between the strength of PSE and immigrants' cultural orientation. Additionally, PSE was identified as a mediator between a stronger orientation toward the mainstream culture and more supportive parenting. The qualitative studies indicated that a reduction in PSE was typical for the initial period of immigration and might be a consequence of a forced orientation toward the standards of the receiving country, which was a consequence of the parents wanting to maintain close relations with their teenage children.

**Discussion:** Overall, the theoretical and methodological quality of the reported studies varied and hence their findings have to be interpreted with caution. Our analysis clearly points to the usefulness of a multifaceted approach to PSE. Further research is needed to understand the mechanisms by which parental self-efficacy may exert a positive effect on the functioning of immigrant families.

## Introduction

Migration can be considered a global and common phenomenon nowadays. According to the UN [Global Migration Data Analysis Centre (GMDAC) International Organization for Migration, 2018], 258 million people, i.e., 3.4% of the world's total population, lived outside the country of their birth or nationality in 2017, which represents a 49% increase compared to the year 2000. Immigration to another country usually implies a series of changes focused on adapting to a new culture. This is a difficult period frequently marked with a negative reception/attitudes in a new place, and a change of language, identity, and values (Miao et al., 2018). Immigrants continuously oscillate between the culture of their birth and their settlement, they constantly face challenges related to maintaining a balance between both cultures, and a pressure to select one of them (Cheah et al., 2013).

In 2017, nearly three quarters (74%) of all international migrants were of productive age, that is, between 20 and 64 years old, whereas children represented 14 percent of migrants [Global Migration Data Analysis Centre (GMDAC) International Organization for Migration, 2018]. Immigrants who are parents, not only face the challenges related to their own acculturation, but also play a special role for their children in the process of adaptation to the new culture (Kim et al., 2014). As a result, tensions related to raising a child (parenting stress) (Lau, 2010; Su and Hynie, 2011) are often coupled with the demands of parents' own cultural adaptation (acculturative stress) (Lee et al., 2016). According to Kim (2018) acculturation stress is a major risk factor for parenting stress. The high level of acculturation stress is often associated with fewer positive parenting practices (Miao et al., 2018), and the general compromised functioning of the family (Lorenzo-Blanco et al., 2016). The experience of successes that build a parent's confidence in his/her own competence as a father/mother (parenting self-efficacy) appears to be particularly supportive in this context (Ochocka and Janzen, 2008), and may be the key mechanism through which levels of acculturation influence parental adjustment (Costigan and Koryzma, 2011).

The concept of parenting self-efficacy (PSE) can be derived from Bandura's (1982) social cognitive theory and is rooted in an agentic perspective on human functioning. PSE is a special case of perceived self-efficacy which, in turn, refers to “people's beliefs about their capabilities to produce designated levels of performance that exercise influence over events that affect their lives” (Bandura, 1994, p. 71). An assumption is made that cognitions of personal efficacy are the core of human agency, and that an individual's performance of specific activities or tasks is linked with the perception of effectiveness of these actions (Bandura, 1997, 2002). Along the same lines, PSE can be broadly defined as the caregiver or parent's confidence about his/her ability to successfully raise children (Jones and Prinz, 2005). According to Coleman and Karraker (2000, p. 13) PSE refers to “parents' self-referent estimation of competence in the parental role,” or to “parents' perceptions of their ability to positively influence the behavior and development of their children,” while Ardelt and Eccles (2001, p. 945) describe PSE as “the parent's belief in his or her ability to influence the child and his or her environment to foster the child's development and success.” So far, research has focused on the role of self-efficacy of fathers and mothers in fulfilling parental functions, their attitudes toward children, understanding of behaviors and emotions occurring in the family, and the psychological adjustment of children (Jones and Prinz, 2005; Wittkowski et al., 2017). A general conclusion is that parents who think that their behavior will have a positive effect on their children engage in positive and supporting parenting strategies (Ardelt and Eccles, 2001; Bandura, 2002; Wittkowski et al., 2017), even in neglected environments (Jones and Prinz, 2005). Furthermore, a high parenting efficacy in mothers is associated with an increased sensitivity and responsiveness to a child (Teti and Gelfand, 1991; Dumka et al., 2010), and with an increased warmth of mothers (Izzo et al., 2000). In the opinion of some researchers (Dumka et al., 2010; Glatz and Buchanan, 2015a), PSE evaluated in a long-term perspective allows us to foresee positive parenting practices in parents of teenagers in terms of controlling, disciplining, and supportive parenting. These factors, in turn, appear to predict lower aggression in children (Jones et al., 2008) and their positive emotional and social development (Page et al., 2010). In contrast, a relationship has been found between lower parenting efficacy and higher dysfunctional parenting characterized by, among others, parental over-reactivity (harsh discipline) and laxness (permissive and inconsistent discipline) (Sanders and Woolley, 2005; Gross et al., 2009). Ardelt and Eccles (2001) found a strong correlation between mothers' parental efficacy beliefs and children's self-efficacy and, indirectly, also with children's academic success, especially in families in particularly difficult situations (i.e., dangerous districts, low income, and low matrimonial support). They also found that parental efficacy beliefs in raising children are a much stronger predictor of children's academic success than parental practices.

There is a growing body of research focused on the construct of parenting self-efficacy and its role in the parent and child's psychological functioning. The way in which individuals perceive themselves as parents is particularly important in the case of immigrants, when a family has to face unique acculturation related stressors in addition to the general parenting stress shared by parents from different cultures (Chung and Epstein, 2014). Explaining the relations between parenting self-efficacy and the process of acculturation may help prevent the consequences of chronic and persistent negative stress (distress). It is surprising that until recently only a handful of studies on the parenting self-efficacy in immigrants have been conducted.

### Current Study

The aim of this systematic review is to structure and summarize the currently available knowledge on the role of parenting self-efficacy in the context of acculturation processes, and in various groups of immigrant parents. The following research questions were addressed:

1. How is parenting self-efficacy conceptualized and measured in studies on immigrants and in the context of culture?
2. What relations can be found among parenting self-efficacy, acculturation processes, and some of the outcomes, as parents' mental health or parenting practices?
3. What, if any, are the effects of promoting parenting self-efficacy in immigrant parents?

## Materials and Methods

### Search Strategy

**Figure 1:**
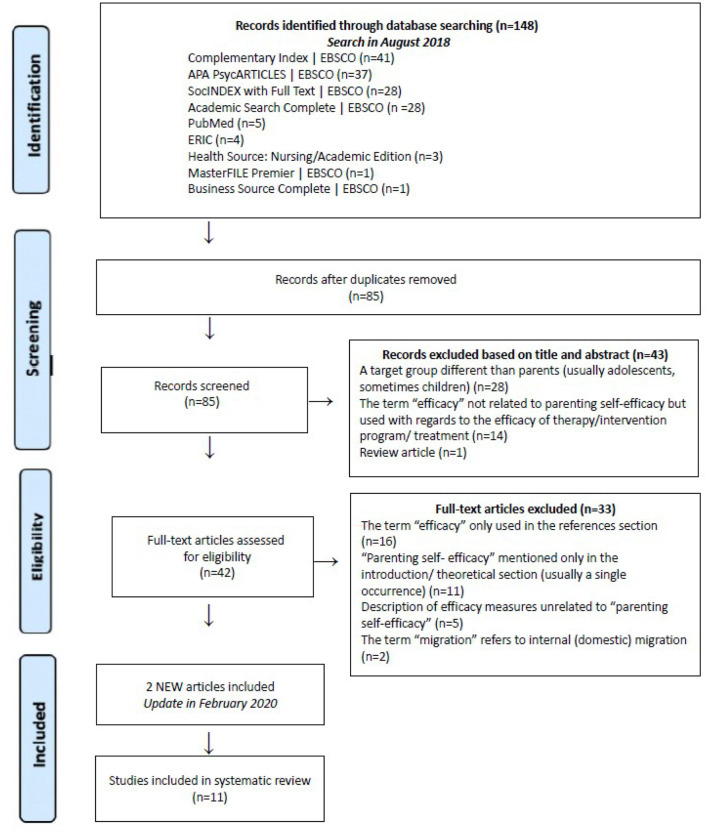
PRISMA flow chart of included studies. Shows the study selection process with numbers excluded at each stage.

The systematic search of eight databases was conducted in August 2018 and updated in February 2020: Complementary Index | EBSCO, APA PsycARTICLES | EBSCO, SocINDEX with Full Text | EBSCO, Academic Search Complete | EBSCO, PubMed, ERIC, Health Source: Nursing/Academic Edition, MasterFILE Premier | EBSCO, Business Source Complete | EBSCO. The search was limited to: (1) original studies, which were (2) full texts (3) on psychology, and were (4) scientifically reviewed. Identification and selection of articles was based on guidelines included in the PRISMA Statement (https://www.prisma-statement.org; see Figure 1).

First, the following keywords, as well as their combinations with AND/OR operators were used: *immigration or migration or migrant or immigrant or refugee or acculturation (abstract) AND self efficacy or confidence or self-esteem or competence (subject terms) AND parenting or parental or maternal (abstract)*.

The differences in the concepts of “self-efficacy,” “confidence,” “self-esteem” and “competence” may be subtle, but they are important to consider (Wittkowski et al., 2017). According to Bandura (1997), parental confidence concerns the strength of a belief about a task, but is not specific in what the strength of the belief is about, whereas parental self-efficacy includes both strength of a belief and an interpretation of capability based on that belief (Wittkowski et al., 2017). Likewise, parental self-esteem is a separate concept. Parental self-esteem is one's judgment of worth as a parent, whereas PSE is one's judgment of personal capability to fulfill the role of a parent (Bandura, 1997). Similarly, parental competence is a separate concept. It refers to the ability to complete a task successfully and efficiently (Pearsall and Hanks, 1998), as does PSE, but it is based on others' perception of how well the task has been completed, rather than a parent's own judgment, as in the case of PSE. Unified and precise terminology would ensure consistency and accuracy. Therefore, the phrase “self-efficacy” or “confidence” or “self-esteem” or “competence” was replaced with “efficacy” in this review. As a result, a combination of key words was formulated as follows: *immigration or migration or migrant or immigrant or refugee or acculturation (subject terms) AND efficacy (all text) AND parenting or parental or maternal (subject terms)*.

### Study Selection

Following the evaluation of full texts by two independent reviewers (JBK, GK), the studies based on the following inclusion criteria were taken into account:

1. Participants: persons self-identifying as immigrants (declared nationality other than the country of residence) who were also parents.
2. A relation to “self-efficacy” understood in line with Bandura's (1997); first of all, studies were included in which the term “self-efficacy” was a key word, but also studies in which measures of self-efficacy were used, e.g., The Parenting Self-Agency Measure, or were a part of a broader tool, such as The Child Adjustment and Parenting Efficacy Scale, Parental Efficacy in the Neighborhood Violence Study or in the Parenting Locus of Control Scale. Studies were only included if “self-efficacy” pertained to a parent.

### Quality Assessment

Quality assessment was performed on the basis of different sets of criteria for quantitative and qualitative studies. Items for evaluation of the quality of quantitative studies Table 2; Supplementary Material were taken from the Quality Index (Downs and Black, 1998), the Epidemiological Appraisal Instrument (Genaidy et al., 2007), and the Evaluation template (Hagströmer et al., 2012), while in the evaluation of three qualitative studies Table 3; Supplementary Material, the criteria for reporting qualitative research (COREQ) were used (Tong et al., 2007). Eventually, 21 control items were used for quantitative studies and 32 control items for qualitative studies. Each item was rated on a three point scale, where 1 was given when the criterion was met, 0—when the criterion was not met (or no data was provided), and “-” when the criterion was not applicable. The quality evaluation was conducted independently by both authors (JBK, GK). High rates of concordance were obtained. For quantitative studies, Cohen's Kappa was 0.9641, 95% CI [0.9146, 1], while for qualitative studies, Cohen's Kappa was 0.9034, 95% CI [0.821, 0.9858]. Inconsistencies were resolved through a discussion until full consensus was reached.

## Results

### Study Selection and Characteristics

The final sample consisted of eleven studies, all of which addressed the issue of parenting self-efficacy in the group of immigrants (Table 1). One of them (9%) was published in 1999, and ten (91%) between 2008 and 2019 (seven (64%)—within the last 4 years).

**Table 1:** Summary of studies included in the systematic review.

**Study**	**Host culture**	**Participants: ethnicity/generation/ family member respondent**	**Children's characteristics**	**Study design**	**Measures of PSE**	**Main information about PSE: definition/key issue/domain (according to Coleman and Karraker, 2000)**
(Yu-wen Ying, 1999) (1)	U.S.	Chinese (68.8% were born in Taiwan)/*N* = 15 (80% mothers and 20% fathers)	School-age children born in the U.S.; all of the parents were fluent in and spoke with their children in Chinese often or all the time; they socialized primarily with other immigrant Chinese (81.3%)	Quantitative; ^*^CS; intervention SITICAF (Strengthening of Intergenerational/Intercultural Ties in Immigrant Chinese American Families); parents participated in three assessment sessions: pre- (immediately before taking the course), post- (immediately after taking the course), and at a 3-month follow-up	Parental Efficacy subscale of the *Parental Locus of Control Scale* (PLOC; Campis et al., 1986)	DEFINITION: No KEY ISSUE: The post-intervention increase in sense of coherence suggests that SITICAF not only increased efficacy in parenting but also the immigrants's general sense of competence in the United States DOMAIN: Domain-specific
(Ali, 2008) (2)	Canada	7 ethno-linguistic communities (Amharic, Arabic, Dari, Mandarin, Somali, Twi and Urdu)/parents who had moved only within the last 5 years/*N* =?; 42 focus groups (consisting of 4–10 individuals) and 42 individual interviews with mothers and fathers	One child below the age of 8 years and the rest above the age of 8 years	Qualitative; semi-structured interview	Significant statements: 1. Parents' Perceptions of their Capacity to Meet Role Obligations: Providing Appropriate Food, Clothing, Housing and Health Care, Supporting Adaptation to School; 2. Engaging in Social and Recreational Activities; 3. Reasons for Reduced Capacity.	DEFINITION: The authors based on theory of Bandura (1997) that a sense of personal efficacy is reflected in the belief that one's actions can lead to the intended outcomes. KEY ISSUS New immigrant parents of young children experience a loss in PSE as a result of their migration to Canada. This injustice also impacts their children, in the short and the long term. DOMAIN: Narrow-domain
(Ceballo and Hurd, 2008) (3)	U.S	Latina/mother – child pairs/30 of the Latina mothers were not born in the U.S, and 17 of the Latina children were not born in the U.S./N=104 (93 biological mothers and 9 legal guardians) Thirty of the Latina mothers were not born in the U.S, and 17 of the Latina children were not born in the U.S.	10-year-old children (grades 4–5); 56 female and 48 male children	Quantitative; ^*^CS	Parental efficacy subscale of the *Neighborhood Violence Study*	DEFINITION: Parental efficacy is one of the four significant parts of parenting strategies (parental efficacy, parental monitoring, parental warmth, and psychological control) KEY ISSUE: Among the Latina mothers, PSE was further diminished with increases in acculturation. Declines in mothers' parenting confidence with acculturation may reflect the competing tensions inherent in simultaneously balancing two sets of cultural norms when making parental decisions. DOMAIN: Domain-general
(Costigan and Koryzma, 2011) (4)	Canada	Chinese/*N* = 177 (96 two-parent: 92 mothers and 85 fathers) Parents who were born outside of Canada and who had been in Canada for at least a year	10–14 years old children	Quantitative; ^*^CS	*Parenting Self-Agency Measure* (Dumka et al., 1996)	DEFINITION: Parental efficacy based on the self-efficacy theory (Bandura, 1997) stipulates that mastery experiences are the most effective way of generating strong feelings of self-efficacy. Parenting efficacy refers to parents' beliefs in their ability to influence their children and the environment in ways that will encourage their children's development (Jones and Prinz, 2005). KEY ISSUE: (1) Relations between Canadian orientation and psychological adjustment were partially mediated by parental efficacy. The more parents were oriented toward Canadian culture, the more efficacious they felt in their parenting, which in turn was associated with better psychological adjustment. (2) Parenting efficacy mediated the relation between higher Canadian orientation and more positive parenting practices (i.e., warmth, reasoning, and monitoring), whereas Chinese orientation was unrelated to parenting practices. Invariance testing suggested that the models were similar for mothers and fathers. DOMAIN: Domain-general
(Lawton et al., 2016) (5)	U.S	Latino—Mexican (88%)/*N* = 74 (47 mothers and 25 fathers)	5–12 years old children (school-age)	Quantitative; ^*^CS	Parental Efficacy subscale of the *Parental Locus of Control Scale* (PLOC; Campis et al., 1986)	DEFINITION: No KEY ISSUE: (1) Parents who feel ineffective in their role as parents are more prone to believe that ADHD symptoms will resolve themselves spontaneously. (2) Parents who endorsed traditional gender roles were more likely to feel limited efficacy as a parent, more likely to believe that parenting and child behaviors are influenced by fate/chance, and more likely to feel unable to control their child's behavior. DOMAIN: Domain-specific
(Yakhnich, 2016) (6)	Israel	Soviet Union (FSU)/*N* = 17 (14 mothers and 3 fathers) living in Israel between one-and-a-half and five years	11–17 years old adolescents	Qualitative; in-depth open interviews	Significant statements: 1. Parental responsibility; 2. Who is the responsible parent: Components of good parenting; 3. Difficulties in fulfilling parental responsibility in immigration; 4. Coping with children's changing behavior.	DEFINITION: Parental efficacy is defined as one's estimation of one's ability to be a competent and successful parent (Coleman and Karraker, 2003) KEY ISSUE Many parents understand that in order to maintain a close relationship with their children, they will have to change, but experience this change as threatening and are not sure whether they will be able to accomplish it. These experiences arouse feelings of uncertainty and helplessness in some parents, thus undermining their sense of parental efficacy and wellbeing. DOMAIN: Domain-general
(Kiang et al., 2017) (7)	U.S	Asian (58) and Latin (153)/*N* = 211 (65% fathers and 35% mothers) the majority were born in the U.S. (78%)	Adolescents (grades 6–12)	Quantitative; ^*^CS	Three questions were developed to assess cultural PSE:: 1. “How much can you do to get your child to practice the customs of your culture of origin?”, 2. “How much can you do to help your child combine Hispanic/Asian and non-Hispanic/non-Asian ways of doing things?”, 3. “How much can you do to instill in your child a sense of what it means to be an ‘American'?”(Kiang et al., 2017)	DEFINITION: PSE defined as the degree to which parents feel capable in their parenting role in general (Abidin, 1995). KEY ISSUE: Among Asian parents cultural PSE associated between higher levels of acculturation conflict and lower levels of perceived parenting competence. DOMAIN: Narrow domain
(Stein et al., 2017) (8)	U.S	Latino/*N* = 172 (94.2% mothers and 2.9% fathers). Participants self-identified as Latina/o/participated in Spanish-language groups/exclusion criteria included focal child not living with parent or active suicidal ideation in parent participant.	(a) Receiving or seeking mental health treatment for their child; (b) 22 years old or younger	Quantitative; ^**^RCT compared a group parental activation intervention with a social support group; intervention MEPREPA—short for “me preparo”/I prepare [MEtas, Preguntar, Escuchar, Preguntar para Aclarar/goals, questioning, listening, questioning to clarify]	*Parent Patient Activation Measure* (PAM; Alegría et al., 2008, 2014)	DEFINITION: A parent's sense of self-efficacy and competence in managing their child's mental health care KEY ISSUE: Intervention MEPREPA helps parents by fostering better communication, teaching parents how best to engage with their providers, and fomenting a sense of efficacy rooted in their role as parents. DOMAIN: Narrow domain
(El-Khani et al., 2018) (9)	Turkey	Syrian refugee/14 mothers and 16 children families with children who had been displaced by the Syrian conflict and were living in Turkey	(a) Child aged 8 or older; (b) child scored 17 or more on the Intrusion or Avoidance scales of the Children's Revised Impact of Events Scale	Quantitative; ^*^CS The teaching recovery techniques (TRT) intervention plus parenting program (three parent skills session) = program “TRT + Parenting”; a pretest-posttest-−1 week before (T1) and 2 weeks after (T2) intervention delivery. No comparison arm was included in the study	Parental efficacy subscale of *The Child Adjustment and Parenting Efficacy Scal**e*** (CAPES; Morawska and Sanders, 2010)	DEFINITION: Self-efficacy is defined as an individual's belief that they can perform a given activity successfully, as well as to the strength of that belief (Bandura, 1997). Parents self-efficacy in dealing with their children's emotional and behavioral disturbances KEY ISSUE The “TRT + Parenting” is an effective brief program for supporting refugee children and their families to reduce signs of PTS, thus enhancing children's mental health, and to increase parents' use of effective strategies and their sense of self-efficacy. DOMAIN: Domain-specific
(Martinez et al., 2018) (10)	U.S	Latino (Mexican 94%)/*N* = 217 families (93% mother and 7% father with a focal youth) aside from one mother, all parents were born outside of the United States	(a) Youth be foreign-born; (b) youth and parent(s) speak either English or Spanish	Quantitative; ^*^CS Families were recruited into the study based on the time in residency (TR) of focal youth, and youth were classified into one of three time-in residence groups	Parenting Efficacy subscale of the *Efficacy parenting* (Capaldi and Patterson, 1989)	DEFINITION: No KEY ISSUE: The most consistent associations between acculturation scale scores and parenting variables involved parental efficacy DOMAIN: Domain-specific
(Malkoff et al., 2019) (11)	U.S	Latinx/*N* = 92 (46 dyads: 46 mothers and 46 fathers) the majority of parents in the sample were born outside of the continental United States (91% of mothers and 96% of fathers)	(a) Child age between 5–13 years; (b) ADHD diagnosis	Quantitative; ^*^CS	Parenting Efficacy subscale of the *Parenting Sense of Competence Scale* (PSOC; Johnston and Mash, 1989)	DEFINITION: Parental efficacy is broadly defined as a parent's beliefs about their confidence and competence in carrying out parenting tasks (Cohen et al., 2015; Heath et al., 2015). KEY ISSUE: (1) As a group, the Latinx parents endorsed feeling efficacious in their role as parents. (2) There were no significant differences between maternal and paternal ratings of parental efficacy. DOMAIN: Domain-general

*Studies are listed by design type*.

* *CS, prospective cohort study*.

** *RCT, randomized controlled trial*.

The sample size ranged from 14 participants in a small pilot study (9) to 217 participants (10). Two studies (18%) included pairs of parents (4, 11), in one study (9%) fathers were the majority (7), six study (55%) samples consisted mostly of mothers (1, 4, 5, 6, 8, 10), and in two studies (18%) the only participants were mothers (3, 9). In one study (9%) dyads of parents took part (11).

Seven of the studies (64%) were conducted in the United States (1, 3, 5, 7, 8, 10, 11), two (18%) in Canada (2, 4), one (9%) in Israel (6), and one (9%) in Turkey (9). The participants were Latin Americans (including Mexicans) in six (45%) studies (3, 5, 8, 10, 11), Chinese in two (18%) studies (1, 4), Asian and Latin Americans in one (9%) study (7), former citizens of the Soviet Union in one (9%) study (6), and Syrians in one (9%) study (9).

In five studies (45%) participants had teenage children (4, 6, 7, 9, 10), three studies (27%) concerned parents of children at a younger school age, i.e., between ca. 5 and 12 years of age (3, 5, 11), in one study (9%) participants were parents of children of a very broad range of ages, from 3 to 22 years old (8), one study (9%) concerned parents of children under 8 years old (2). In three studies (27%) participants were parents of children with some mental health problems like ADHD, PTS, depression or anxiety (8, 9, 11).

Nine (82%) studies (i.e., the majority) were quantitative (1, 3, 4, 5, 7, 8, 9, 10, 11) and two (18%) were qualitative (2, 6). Three studies (27%) assessed interventions/parenting programs for immigrants (1, 8, 9).

## Synthesized Findings

### Conceptualization of Parenting Self-Efficacy and Measurement Considerations

There are 34 measures of PSE and related concepts currently available, all based on self-report. Noteworthy, since 1970, none have been widely adopted, indicating that measures might have been developed for specific applications (as summarized in Wittkowski et al., 2017) and with differing underlying conceptual assumptions. According to Coleman and Karraker (2000), at least four approaches to conceptualizing (and studying) PSE can be distinguished: general or trait, domain-general, domain-specific, and narrow domain/task specific. “General PSE” represents a relatively stable personality trait of broad applicability to diverse domains of human functioning, with parenting representing only one such domain (Coleman and Karraker, 2000). As pointed out by Wittkowski et al. (2017), measures elaborated in line with this conceptualization of PSE assess overall beliefs of efficacy in the parenting role, with items not linked to any specific parenting task (e.g., “What I do has little effect on my child's behavior.” Campis et al., 1986). Noteworthy, this approach significantly departs from the original Bandurian conceptualization of self-efficacy as pertaining to highly task specific efficacy cognitions. None of the papers under our review adopted this particular, most global stance.

Within “domain-general” (also referred to as “global”) approach, parenting self-efficacy is considered to be conceptually distinct from efficacy beliefs pertaining to other domains, but at the same time is assessed on the basis of global competency expectations that are not linked to particular parenting tasks (Coleman and Karraker, 2000) (e.g., “I feel sure of myself as a parent,” Dumka et al., 1996). The focus is broadly on the extent to which a parent feels competent in the parenting role, without referring to specific parenting tasks or a particular domain of parenting (Jones and Prinz, 2005). A somewhat different perspective is assumed by Wittkowski et al. (2017) who consider the “domain-general” measures as referring to everyday reality of parenting, while not specifying the concrete parenting tasks or activities (e.g., “I know good parenting tips that I can share with others,” Freiberg et al., 2014). Measurement tools inspired by this conceptualization of PSE, although less sensitive to the tasks that are faced by a parent of a child of a specific age, can be quite universal, i.e., suitable for a wide range of child ages (Crnčec et al., 2010). “Domain- general” approach was used in four studies included in our review (Ceballo and Hurd, 2008; Costigan and Koryzma, 2011; Yakhnich, 2016; Malkoff et al., 2019). In the study by Ceballo and Hurd (2008) parenting efficacy was assessed on the basis of three items, where parents were asked to indicate how often they felt “overwhelmed by parenting demands,” “as if you were not in control as a parent,” and “stressed and worried about the demands of parenting in your neighborhood.” In contrast, Costigan and Koryzma (2011) used the *Parenting Self Agency Measure* (Dumka et al., 1996). This scale is designed to evaluate confidence in the parenting role, feelings of helplessness in the face of challenging child behavior, and the degree of parenting effort and persistence (e.g., “I know I am doing a good job as a mother/father”). In the study by Malkoff et al. (2019), mothers and fathers completed the Parenting Efficacy subscale of the *Parenting Sense of Competence Scale* (PSOC; Johnston and Mash, 1989) (e.g., “I honestly believe I have all the skills necessary to be a good parent to my child”).

“Domain-specific” (also referred to as “task-related”) PSE involves combining task-specific measures of self-efficacy into a single measure of self-efficacy within the broader domain of parenting (Bandura, 1997). According to Jones and Prinz (2005), “domain-specific” PSE is similar to domain-general PSE in assessing PSE globally, but the items themselves are task-specific (e.g., related to childrearing activities such as toilet training, learning readiness tasks, or caring for a sick child). Task-related PSE measures cover a wide range of parenting domains such as discipline, warmth, meeting instrumental needs, and supervision/monitoring to yield a summarized index of PSE. This approach offers greater sensitivity to specific tasks and ages, leading to greater predictive validity of tool than domain-general PSE measures (Wittkowski et al., 2017). Bandura (1997) argued that PSE is most accurate when assessed with domain-specific measures. The “domain-specific” approach was used in four studies included in this review (Yu-wen Ying, 1999; Lawton et al., 2016; El-Khani et al., 2018; Martinez et al., 2018). Two of them (Yu-wen Ying, 1999; Lawton et al., 2016) used the Parental Efficacy subscale of the *Parental Locus of Control Scale* (PLOC; Campis et al., 1986). This measure assesses the parental sense of being effective in the role of a mother or a father (e.g., “When something goes wrong between me and my child, there is little I can do to correct it.”). In the third study (El-Khani et al., 2018), the Parental efficacysubscale of *The Child Adjustment and Parenting Efficacy Scale* (CAPES; Morawska et al., 2014) was used. CAPES assesses parents' self-efficacy in dealing with their children's emotional and behavioral disturbances (e.g., parent's confidence when the child seems to be unhappy/sad or argues/fights with other children, brothers, or sisters). In the last study by Martinez et al. (2018), the Parenting Efficacy is one of the three scales of the *Effective Parenting* (Capaldi and Patterson, 1989), and consists of items reflecting general past month use of effective parenting strategies.

Finally, “narrow-domain” (also referred to as “task-specific”) approach focuses on parents' perceptions of their own competence with respect to specified tasks within the domain of parenting, like for instance, identifying physical illness in their children (Coleman and Karraker, 2000). In contrast to “domain-specific” conceptualization of PSE, the focus here is on a single parenting domain such as discipline, promotion of learning, or communication (Jones and Prinz, 2005). As noted by Wittkowski et al. (2017), in this approach one specific aspect of the parenting role is assessed, and the items should be all task-specific, age-specific, and situation-specific. Among the papers included in our review, three out of eleven studies used measures of PSE in relation to specific tasks, such as meeting the child's basic needs and navigating through the process of adaptation to new environment (Ali, 2008), managing child's health (Stein et al., 2017), and enhancing adolescent cultural identification (Kiang et al., 2017). Various methodological approaches were applied to measuring narrow-domain PSE, from relying on semi structured interviews on a number of task specific topics, both with individual participants and in focus groups (Ali, 2008), via a short survey comprising three questions about parental cognitions of their task- specific efficacy (Kiang et al., 2017), to a standardized 13-item Parent Patient Activation Measure (PAM) used at three distinct time points (Stein et al., 2017). Detailed information on each of the included studies is presented in Table 1.

### The Concept of “Parenting Self-Efficacy” and Culture

#### Parenting Self-Efficacy and Acculturation

In the studies included in this systematic review, the role of PSE is analyzed mainly in the context of the direction of acculturation, which is understood as an orientation toward one of the cultures (Costigan and Koryzma, 2011), and in relation to acculturation conflict, that is, a conflict between a parent and a child concerning cultural issues (Kiang et al., 2017). Possible links to parental psychological adjustment and parenting practices are sought for.

Results of the study by Costigan and Koryzma (2011) suggest that an orientation toward the culture of the receiving country is associated with a stronger parenting efficacy, better parental psychological adjustment, and more positive parenting practices. These results are consistent with other studies, which have demonstrated that a stronger orientation toward a new culture is associated with better adaptation (Birman and Taylor-Ritzler, 2007; Hwang and Ting, 2008). Parents, who feel more efficient in their parental role, undertake more active and persistent efforts to overcome difficulties, and this, in turn, results in more efficient parenting (Dumka et al., 1996; Bandura, 1997), with possible positive effects for mothers' and fathers' mental health. In contrast, the results obtained by Ceballo and Hurd (2008) show that in the case of mothers the sense of parenting efficacy decreased with increases in acculturation. A drop in parents' trust in their parenting skills may result from tensions caused by problems with making decisions associated with balancing between two sets of cultural values and standards. These results should be treated with caution due to certain methodological limitations of the study. Specifically, the measurement of the level of acculturation may raise some doubts, as it was based solely on mothers' answers to three general questions: (1) was the mother born in the USA, (2) was the child born in the USA, (3) did the mother prefer to answer the questions in English, when she could choose between Spanish and English. In still another study, Malkoff et al. (2019) indicated that Latinx parents of children with ADHD, who were more acculturated to traditional Latinx culture than mainstream U.S. culture, felt efficacious in their role as parents and no significant parental gender differences were found in ratings of this variable. However, mothers reported higher levels of parenting stress than fathers. This is probably due to the fact that within the traditional Latinx culture the burden of caregiving responsibilities is placed more on mothers than fathers (Barker et al., 2010). Furthermore, the experience of stress may be even more pronounced in mothers with limited financial and psychological resources to meet their children's needs (Nomaguchi and House, 2013).

Notably, in the study by Costigan and Koryzma (2011) possible links between acculturation, parenting efficacy, parental psychological adjustment and parenting practices were examined for both mothers and fathers. Parents' orientation to the ethnic culture (Chinese) was not statistically significantly associated with either parenting efficacy or with psychological adjustment (Costigan and Koryzma, 2011). At the same time, parenting efficacy partially mediated the relationship between the Canadian orientation (orientation toward the new culture) and adjustment (psychological and parental) in both mothers and fathers. Furthermore, parenting efficacy was also found to mediate the relation between higher Canadian orientation and more positive parenting practices. The authors suggest that the key reason for which higher orientation to the receiving country is associated with more positive parenting outcomes may be higher parental confidence experienced by mothers and fathers who are more involved in the new environment. These results point to possibly diverse roles of the new culture and the ethnic culture for parenting self-efficacy and psychological adjustment, and justify the value of assuming a bidimensional evaluation of acculturation (Berry, 2003).

The more immigrant parents perceive that they and their children experience acculturation conflict, the less competent they feel as parents in general (Yakhnich, 2016; Kiang et al., 2017). It is possible that any form of a conflict, whether related to acculturation or not, reduces the sense of competence and the sense of self-esteem in parents (Glatz and Buchanan, 2015b). Furthermore, acculturation conflict can be regarded as one of the sources of stress in family life that has a direct bearing on parental attitudes in immigrants and ethnic minorities (Telzer, 2010). In the study by Williams et al. (2017) it was found that experiencing high levels of acculturation stress was associated with negative parenting practices and a higher number of family conflicts when social support was insufficient.

#### Cultural Parenting Self-Efficacy

Having the immigrant parents in mind, Kiang et al. (2017) introduced a more specific term, that is, “cultural parenting self-efficacy” (Kiang et al., 2017), which they define as the extent to which parents believe that they can effectively pass to their children knowledge, values, and pride concerning the culture of their country of origin or residence. Contrary to the general perceived parenting competence (Coleman and Karraker, 2000), the concept of cultural PSE focuses on parents' targeted perceptions of their own ability to directly influence their children in the context of socialization within three different areas of culture—heritage, mainstream, and bicultural (heritage and mainstream). As mentioned before, conceptually it is close to domain-specific PSE.

In Kiang et al. study 2017, parents from Latin America achieved a much higher average level of heritage and mainstream cultural PSE compared to parents from Asia; however, it was only in Latin American parents that all three cultural dimensions of PSE (heritage, mainstream, and bicultural) were associated with a higher perceived general parenting competence. Notably, other studies show that orientation toward the mainstream culture is more adaptive for immigrants (Ryder et al., 2000; Hwang and Ting, 2008; Costigan and Koryzma, 2011), and this suggests that insufficient endorsement of the mainstream culture may result in psychological maladjustment, such as depressive symptoms and a lack of bicultural efficacy, or in other words, more bicultural management difficulty. However, there have been inconsistent findings across studies (e.g., Ryder et al., 2000; Birman and Taylor-Ritzler, 2007), and even across two parents in the same family (Costigan and Koryzma, 2011).

In addition, cultural PSE turned out to be a buffer against reduction of general parenting competence under high levels of parent-child acculturation conflict, but only for Asian Americans, and not for Latin American parents (Kiang et al., 2017). Asian parents, who felt more confident in passing Asian values to their children (cultural PSE), perceived their general parenting competence as higher than parents who felt less efficient in that respect. It is likely that strong cultural values that these parents may have enable them to cope with the acculturation stress and to perceive the acculturation conflict between them and their children as temporary, and hence maintain a positive perception of themselves as parents.

### The Effects of Enhancing Self-Efficacy in Migrant Parents: Data From Intervention Studies

Considering the mediating role of self-efficacy between knowledge and behavior (e.g., Bandura, 1982, 1997), the impact of group interventions affecting parenting skills on parental self-efficacy is an area of great importance (Wittkowski et al., 2016). However, the number of programs and specialists dedicated to parents struggling with adaptation to a new cultural context is scarce. Through a higher awareness and understanding of relations between acculturation, parenting efficacy and psychological adjustment, the specialist community could support immigrants at an individual and at a systemic/family level. Yet, research evidence in this area is, unfortunately, still very limited. For the purposes of this systematic review, we have managed to identify only three studies addressing the issue of interventions designed for immigrant parents and aimed at managing acculturation stress.

The first of the three studies, conducted by El-Khani et al. (2018), is quite specific as it addresses the issue of feasibility of an intervention programme designed for Syrian refugee families. It is an extension of the *Teaching Recovery Techniques* (TRT) program of the Children and War Foundation (Yule et al., 2013) as an aid to professionals working with children aged 8 years and older affected by wars/armed conflicts (also disasters) and displacement. The original TRT focuses not on symptom reduction as such, but rather on teaching children practical skills and techniques to cope with psychological consequences of war and violence. Notably, its aim is not only to help children deal with past and present difficulties but also to prepare for the future and prevent mental health problems. The TRT+Parenting intervention was developed to specifically address the unmet needs of parents with respect to supporting and taking care of their children in the context of war and displacement (El-Khani et al., 2016a,b). A significant decrease in the quantity and intensity of behavioral problems in children (measured with the CAPES scale) can be regarded as a strong indicator of positive changes. In the parents' behavior, a significant reduction in excessive indulgence and reactivity, and a significant increase in parenting efficacy were noted, and this indicates that additional parenting skill sessions added to the TRT program were successful in increasing parents' competence and in supporting them to build context-sensitive skills to provide better care for their children. The exceptionally high attendance and programme completion ratios point to the program's feasibility and indicate that families are willing to participate in interventions of this kind because they need them.

The SITICAF *(The Strengthening of Intergenerational/Intercultural Ties in Immigrant Chinese American Families*) program from the Yu-wen Ying study 1999 is aimed at removing the intergenerational and intercultural gaps in families of American immigrants from China by increasing parents' awareness in this area, promoting intercultural competence, improving parental control and development of efficient parenting skills, particularly in communication, and by enabling parents to better cope with intergenerational/intercultural conflict. The results of the study suggest a significant increase in parents' sense of responsibility, quality of intergenerational (parent-child) relationship, as well as general sense of parenting competence.

In their randomized controlled trial (RCT) with the use of MEPREPA intervention (an abbreviation of “me preparo”/I am ready MEtas, Preguntar, Escuchar, Preguntar para Aclarar/aims, asking questions, listening, asking to receive explanations), in a group of Latino/a families, Stein et al. (2017) show that parental stress probably leads to a low self-efficacy that contributes to low activation of parents in seeking mental health services for their children with ADHD symptoms. As a result, parents can be less effective in providing their child with access to necessary assistance, and less optimistic in perceiving their abilities to support beneficial changes. The results of the trial show that parents with more symptoms of depression and a higher level of stress benefited more from the MEPREPA as compared to the control group (support group). Furthermore, the intervention was more effective in the case of parents whose children had more pronounced symptoms.

The results of all three studies clearly show that interventions focused on enhancing various aspects of parental self-efficacy in immigrant parents bring about positive outcomes in the context of acculturation challenges. Notably, there is a significant connection between increased sense of parental control and a positive parent-child relationship, which in turn is associated with higher self-esteem in the child (Yu-wen Ying, 1999). It appears that promoting parenting self-efficacy and the use of more effective parenting practices is beneficial for all members of the family (Parra Cardona et al., 2009).

### Limitations of the Analyzed Studies and Future Directions

The analyzed studies have many strong aspects, but they are also characterized by certain recurring limitations, indicating directions for future studies.

First, the lack of longitudinal studies (due to financial or legal issues) makes it impossible to evaluate the direction of a relationship between the analyzed cultural and contextual variables and parenting self-efficacy. The results were mainly based on correlational (Costigan and Koryzma, 2011; Kiang et al., 2017) and cross-sectional (Martinez et al., 2018) data, thus no causal conclusions can be drawn. Furthermore, due to the lack of follow-up studies verifying the effectiveness of the interventions, it is not possible to assess if the reported changes in PSE will be sustained over time (El-Khani et al., 2018; Martinez et al., 2018).

Second, the results may be biased as they were mainly based on self-report measures of PSE. Just one study by Ceballo and Hurd (2008) used children's assessment of the parental monitoring. This is a more general problem in research on parenting. Future studies should consider incorporating multimodal forms of assessing parenting beliefs and behavior to determine if the current pattern of findings remains consistent. Where possible, future studies should make use of the triangulation of methods (Martinez et al., 2018), for example combine cross-sectional reports within each dyad of parents (Malkoff et al., 2019) with children's evaluation of their parents' parenting practices (Yu-wen Ying, 1999; Costigan and Koryzma, 2011; Kim et al., 2014; Kiang et al., 2017) or direct observations of parenting behaviors and parent-child interactions (Malkoff et al., 2019), in order to obtain more objective measures of parenting.

Third, the study samples were formed by enrolling volunteers. This sampling method may raise some doubts, as families that volunteered for the study may not be representative of the studied populations (Yakhnich, 2016; El-Khani et al., 2018).

Fourth, the national minorities most frequently studied are Asian Americans and Latin Americans, who, however, represent very diversified ethnic groups. Thus, due to a relatively small size of studied subgroups (Yu-wen Ying, 1999; Ceballo and Hurd, 2008; Kiang et al., 2017), collected data cannot be used to analyze possible intragroup differences, which in some cases can be even more important than differences between studied groups of Asians and Latin Americans (Bandura, 2002; Kiang et al., 2017; Martinez et al., 2018; Malkoff et al., 2019). Furthermore, the homogeneity of the sample limits possible generalization of the results (Yu-wen Ying, 1999; Malkoff et al., 2019). With a small sample size, randomized projects or multidimensional analyses testing the intervention effectiveness are not possible (Yu-wen Ying, 1999; Yakhnich, 2016; Martinez et al., 2018). Studies of other ethnic groups and in other cultural contexts are clearly lacking.

Fifth, except for Martinez et al. and colleagues' study 2018, there is no information on the cultural adaptation of measures for the studied populations. Unfortunately, a common practice in many studies is to limit adaptation process to the phase of translation of questionnaire items without any empirical attempt to check their suitability to new cultural contexts (Martinez et al., 2018).

Sixth, mainly mothers (Yu-wen Ying, 1999; Yakhnich, 2016; Martinez et al., 2018) or only mothers (Ceballo and Hurd, 2008; Stein et al., 2017) participated in the analyzed studies. In the intervention dedicated to Syrian parents none of the fathers participated, although they were invited to the program (El-Khani et al., 2018). Dyads of parents participated in one study (Malkoff et al., 2019). The sample studied by Kiang et al. (2017) was exceptional, as 65% of participants were fathers. In many contemporary cultures, both mothers and fathers are responsible for raising children (Chao and Tseng, 2002). Therefore, in future works it would be worthwhile to focus on possible differences and similarities in parents of both genders. Interestingly, the results of one study point to gender differences in PSE, i.e., to the association of PSE with actual parenting practices of mothers, but not of fathers (Glatz and Buchanan, 2015a), whereas in another study (Malkoff et al., 2019) no significant parental gender differences were found in ratings of parenting efficacy.

Furthermore, the study conducted in the area of armed conflict (El-Khani et al., 2018) is worth noting. For safety reasons, it was remotely controlled from the United Kingdom. The researchers could not go in person to the area where the study was conducted due to continued military actions in that area; therefore, four local teachers went to Istanbul where they were trained by a trainer from the UK. Then they returned to their local community and conducted the intervention in the school in which they taught. They could communicate with researchers in the UK through mobile applications, such as Skype or Whatsapp (El-Khani et al., 2018). This may point to peculiar challenges related to studying parenting self-efficacy in immigrants under diverse life circumstances.

## Discussion

The aim of this systematic review was to summarize available data on the conceptualization, measurement, and the role of parenting self-efficacy in the context of acculturation processes, and in various groups of immigrant parents.

The results of quantitative studies qualified for the review point to a multi-dimensional context of PSE. The results of the study by Costigan and Koryzma (2011) demonstrate that a stronger orientation toward Canadian (mainstream) culture may increase the PSE level, because parents have knowledge and skills increasing their confidence as parents in a new, international environment (Costigan and Koryzma, 2011). In the study conducted by Ceballo and Hurd (2008) amongst Latin American mothers, the PSE level decreased as the level of acculturation toward the American (mainstream) culture increased. The decrease in the confidence of mothers in their parental competences may reflect the stress related to finding a balance between the standards of the two cultures when making parental decisions. The authors conclude that protective aspects of a strong orientation toward the inherited culture result from a greater support and understanding from other Chinese parents in the same situation (Costigan and Koryzma, 2011). This potential support can, however, be insufficient with regard to parental challenges that appear when parents are strongly oriented toward the inherited culture. For example, parents that maintain a high level of involvement in Chinese culture, that is, prefer Chinese language, entertainment, identity and social relations, may experience a greater detachment from their children, when compared to parents who are less oriented toward Chinese culture (Costigan and Dokis, 2006), and this reduced sense of relationship may weaken the parenting efficacy (Costigan and Koryzma, 2011). Additionally, in this study PSE mediated the relationship between a stronger Canadian orientation and more positive parenting practices (warmth, reasoning, and monitoring). Furthermore, orientation toward Chinese culture did not appear to be associated with parenting practices, and these results were independent of the parent's gender (Costigan and Koryzma, 2011). In the study by Kiang et al. (2017), concerning a more detailed, cultural aspect of PSE, Latin American parents were characterized by a much higher level of cultural PSE (inherited and American) than Asian parents. Cultural PSE was associated with a higher perceived level of a general parenting competence, but only in Latin American families. The more Asian and Latin American parents perceived that they and their children were in acculturation conflict, the less competent they felt as parents (Kiang et al., 2017). Faced with the parent—child acculturation conflict, Asian parents who felt confident in passing traditional values to their children were characterized by a higher level of general parenting competence than parents who felt less efficient in that respect. The obtained outcomes may result from the fact that Latin Americans are more open in communication with their children in relation to cultural difference, and in passing to them social competences depending on a given culture, and this positively influences their general parenting competences. On the other hand, for Asian Americans who are associated with the stereotype of a “forever foreigner” and unassimilable (Tuan, 1998), the conflict between them and their children concerning cultural issues may have a more important impact on their perception of parenting competences than their cultural PSE alone. A generation of studied people is also worth noting, as the majority of parents from Latin America were second-generation immigrants, while parents from Asia were equally divided between the first and the second generation (Kiang et al., 2017). It should be emphasized that this is only the perspective of parents. The results could have been different if they had included data collected from children.

On the other hand, in the study by Lawton et al. (2016) self-efficacy was associated with parents' conviction that in the case of their children symptoms characteristic for ADHD would resolve themselves spontaneously. The more inefficient the parents felt, the stronger their belief that the problematic behaviors of their children would disappear spontaneously. Additionally, parents valuing the traditional division of roles more frequently felt that their efficacy was limited, that is, they were not able to control the behavior of their children. Apart from that, they also more strongly believed that their and their children's behavior depended on fate/chance. The value of the traditional division of roles appears to be in contrast to the values of American (mainstream) culture. It is possible that the Latin American parents, who value the traditional division of roles, feel uncertain about how to use the traditional parenting practices within the framework of American culture, and thus feel less efficient and in control as parents. In the study by Malkoff et al. (2019), in turn, the Latinx parents of children with ADHD felt efficacious in their role as parents. This is consistent with Weinberger et al. (2018) study finding that mothers of ethnic minority children with ADHD reported greater parental efficacy than mothers of European American children with ADHD.

It is worth noting that all these results were obtained in studies with correlational designs, and therefore no causal relations can be inferred.

The review also included two qualitative studies, which imply that Canadian immigrants who have small children experience a decrease in the PSE level, particularly in the initial stage. The parents' observation is that migration has a negative impact not only on them, but also on their children, in a short- and long-term perspective (Ali, 2008). Many parents from the former USSR living in Israel (Yakhnich, 2016) feel a clear conflict between “old” and “new” caused by their exposure to new social standards. They understand that in order to maintain a close relationship with their teenage children, they will have to change, but this appears dangerous to them, and they are not sure whether they will succeed. In some parents, this results in uncertainty and helplessness, undermining their parenting self-efficacy.

Another important group of studies that was included in the review are studies focusing on the effectiveness of interventional programs dedicated to immigrants. It is worth noting that immigrant parents may be more open to interventional programs focusing on parental roles than to those focusing directly on general immigration or psychological adjustment (Parra Cardona et al., 2009). The studies included in the review indicate that the interventional programs result in a reduction of excessive indulgence and hyperreactivity in parents' behavior, while the parenting efficacy (El-Khani et al., 2018), parents' sense of responsibility and competence, and the quality of intergenerational (parent-child) relationships (Yu-wen Ying, 1999) increase. It is worth noting that interventions dedicated to immigrant parents should take into account the cultural aspect, and be embedded in contemporary reality (Coard et al., 2004; Kiang et al., 2017). Furthermore, a decrease in the acculturation conflict between parents and children may directly increase the sense of parenting competences, as well as increase the general closeness in the family and improve communication (Parra Cardona et al., 2009), thus increasing the overall effectiveness of a therapy or an intervention (Kiang et al., 2017).

### Summary of Evidence

“Parenting self-efficacy” in immigrant parents has been conceptualized and measured within the framework of domain-general, domain-specific and narrow domain approaches (Coleman and Karraker, 2000). The studies included in this review differ significantly in methodology. The analyzed studies were: (1) quantitative studies, in which PSE was assessed on the basis of domain-general and domain-specific measures (questionnaires focused entirely or partly on PSE); (2) qualitative studies in the form of semi-structured interviews and observational methods, and (3) studies measuring the effectiveness of interventions dedicated to immigrant parents.

Mixed findings were found as to the relationship between PSE and the direction of acculturation. The results of one of the studies under review indicate that a stronger orientation toward the mainstream culture was associated with a higher level of PSE (Costigan and Koryzma, 2011) and PSE mediated the relationship between a stronger orientation toward the mainstream culture and more positive parenting practices (Costigan and Koryzma, 2011). On the contrary, the study by Ceballo and Hurd (2008) showed that orientation toward the mainstream culture was associated with reduced PSE. Similarly, Malkoff et al. (2019) indicated that Latinx parents of children with ADHD from low-SES backgrounds and more acculturated to traditional Latinx culture than mainstream U.S. culture felt efficacious in their role as parents. The study by Kiang et al. (2017) shows that in the face of parent-child acculturation conflict, Asian parents who felt confident in passing on traditional values to their children, were characterized by a higher level of general parenting competence than parents who felt less efficient in that respect, and that the more the parents noticed that they and their children were experiencing a conflict on issues associated with acculturation, the less competent they felt as parents (Kiang et al., 2017). At the same time, the qualitative studies indicated that a reduction in PSE was associated with an initial period of immigration (Ali, 2008), and might be a consequence of a forced orientation toward the standards of the host country resulting from a wish to maintain close relations with teenage children (Yakhnich, 2016). Furthermore, the study by Lawton et al. (2016) shows that the more inefficient parents feel and the stronger their belief that their and their children's behavior depends on fate/chance, the stronger their conviction that the problematic behavior of their children will resolve itself spontaneously.

The studies concerning interventions (“TRT+Parenting” programme, SITICAF, MEPREPA) show that actions of this kind increase parenting efficacy (El-Khani et al., 2018), increase parents' sense of responsibility, and improve the quality of the parent-child relationship (Yu-wen Ying, 1999). Parents with higher depressive symptoms, greater parenting stress, and whose children had more serious symptoms benefited more from the intervention as compared to the controls (support group) (Stein et al., 2017). Generally, high attendance and program completion ratios indicate that families are willing to participate in interventions of this kind because they need them.

### Quality Assessment

The results of quality assessment were within the range of 31.6–100%, with an average of 86.2% for the quantitative studies, and 53.3% for the qualitative studies; therefore, the average for both types of studies was 69.8% Tables 4, 5; Supplementary Material.

The quantitative studies scored highly in the areas of the hypothesis/aim/objective of the study, exposure variables/intervention(s), design and appropriateness to the hypothesis/aim, main outcomes to be measured, characteristics of study participants, important covariates and confounders, main findings, statistical parameters, conclusions, statistical tests, and methods for assessing the outcome variables. The lowest scoring item was the inclusion of characteristics of subjects lost after enrolment in the study or subjects not participating from the eligible population, and the inclusion of information on details of the sample size. Only one study was a cohort one (Stein et al., 2017), in which it was possible to randomize subjects into intervention groups. In this study a research assistant was blind to intervention status when conducting a baseline interview prior to starting a group and then during data collection and analysis. These two questions about the use of techniques of randomization and blinding were not obligatory during quality assessment. One study by El-Khani et al. (2018) scored 100%.

The qualitative studies scored highly on researchers' credentials, experience and training, the method for approaching participants, and sample description. None of the studies scored on items concerning participants' knowledge of an interviewer, presence of non-participants, repeated interviews, transcripts returned, and participants' feedback on the findings.

### Limitations and Future Directions

Conclusions drawn from this systematic review, like other works of this type, are strictly related to search criteria. One of the limitations is that our search was confined to papers published in English, which potentially leaves out the results of studies available in other languages. This may also partly explain why most of the included studies pertain to Latinx or Chinese immigrants in North America. In addition, our search of databases was performed with a focus on parenting self- efficacy construct as such. Potentially different results could have been obtained if the search had been primarily guided by parenting-self efficacy measurement. Despite these limitations, our review clearly shows that focusing on parenting self-efficacy in immigrants may valuably contribute to the existing knowledge on the potential consequences of acculturation for families with children. This issue is of utmost importance due to the huge scale of migration that concerns parents and children, and to the potentially crucial role of PSE in promoting mental health. Further research with more diverse immigrant groups and a wider range of receiving cultures is definitely required.

### Implications for Clinical Practice and Therapy

The analyzed studies contain many implications for clinical practice and therapy. One of the main issues is stress associated with migration, which is a strong risk factor for dysfunctions in families (Smokowski et al., 2008; Kiang et al., 2017). Therefore, knowledge about the acculturation process (e.g., a direction of its orientation, level of acculturation, a parent-child conflict) in immigrant parents may help to identify people at risk of decreased parenting self-efficacy, and thus, possibly, also of lower indices of mental health and parental adaptation (Costigan and Koryzma, 2011). Investing in improvement in the PSE level leads to improvement in the quality of life of parents and children, particularly those living in difficult conditions. It can also have a protective effect against risk factors associated with higher stress or poverty (Jones and Prinz, 2005). Immigrant parents that face challenges associated with involvement in the new culture are at a higher risk of using less effective parenting practices (Costigan and Koryzma, 2011). It is important to encourage immigrant parents to search for a form of support suitable for their needs (independent of or related with the promotion of biculturality). National and local programs are required, focusing on increasing the confidence of immigrant parents in their skills related to efficient parenting. These interventions should particularly take into account problems of immigrants related to cultural identification of children and parents (Schwartz et al., 2010), as well as cultural beliefs and values (Lawton et al., 2016).

Professionals who have contact with immigrant parents should remember that they and the families they work with are frequently in a *power relation* for historical, economic, political, and social reasons, and due to cultural characteristics of their societies. Remembering that may help them to understand that barriers hindering immigrant families from becoming involved in programs or interventions dedicated to them are enrooted in power relations, and are not mainly an effect of cultural and language differences. They do not merely result from lack of time or insufficient institutional support. It is important for the specialists to be able to accept and face the limitations of their own knowledge about children and families they work with. The understanding of own biases could help them resist the tempting idea that they possess some “universal truths” (Ali, 2008). These specialists must bear in mind that they are responsible not only for what they do, but also for what they do not do (Noddings, 1984, 2005). Each action or omission may contribute to the resistance of families with which they work, or to promoting unfair attitudes of power (Mac Naughton, 2005). For this reason, training and workshops for specialists providing direct assistance to immigrant families, that is, for employees of institutions providing care for children in kindergartens and schools, health care professionals, and social workers, are of importance (Ali, 2008). Another equally important method for stopping a decreasing level of PSE in immigrant parents is introduction of their narration and reconstruction of their experiences, with a particular emphasis on their strengths, into a general social awareness in which they currently live (Ali, 2008). This can be achieved by popularizing scientific research concerning this area, media projects (documentary films and publications, social campaigns), and/or organizing cultural and educational events (festivals, reviews, meetings, workshops).

## Conclusions

Our analysis demonstrated that studies focusing on “parenting self-efficacy” in immigrants are scarce. The studies included in the review are characterized by a high level of variability in both theoretical and methodological approaches. Likewise, in general, there are many inconsistencies in the literature about “parental self-efficacy.” This can create difficulties in comparing and integrating knowledge concerning PSE, hampering progress in understanding how PSE is formed, operates, and how it can be modified. However, this variability also creates a space to analyze the construct from many perspectives. With a holistic understanding of the parenting factors, it is more likely that researchers and clinicians will be able to develop appropriate intervention strategies and adapt existing interventions to better meet immigrant parents' mental health needs. The review aimed to provide the currently available knowledge for researchers and clinicians working with families of immigrants to help guide their choices by clarifying terminology, and reviewing the available measures. The aim of this systematic review was to structure and summarize up-to-date information on the role of parenting self-efficacy in the context of acculturation processes, and in various groups of immigrant parents. It is important that when parents have conviction and belief in their own abilities, the quality of parenting can be optimized and the role of being a parent can become as pleasurable as possible.

## Author Contributions

Both authors contributed to the review design, search criteria, and writing of the paper.

## Conflict of Interest

The authors declare that the research was conducted in the absence of any commercial or financial relationships that could be construed as a potential conflict of interest.
