# Supplementary material for: Parenting Self-Efficacy in Immigrant Families—A Systematic Review
Source: Front Psychol. 2020 May 26;11:985. doi: 10.3389/fpsyg.2020.00985 (PMC7264399; doi:10.3389/fpsyg.2020.00985)
Supplement: Supplementary file 1 [file Data_Sheet_1.doc]

Appendix 1: Quality assessment items - quantitative study

| No | Item |
| --- | --- |
| *Study Discription* | |
| 1 | Is the hypothesis/aim/objective of the study clearly described? |
| 2 | Are all the exposure variables/intervention(s) clearly described? |
| 3 | Is the study design clearly described and appropriate to test the hypotheses/aim? |
| 4 | Are the main outcomes to be measured clearly described? |
| 5 | Are the characteristics of participants in the study clearly described? |
| 6 | Are the important covariates and confounders described in terms of individual variables? |
| 7 | Have the characteristics of subjects lost after entry into the study or subjects not participating from among the eligible population been described? |
| 8 | Are the main findings of the study clearly described? |
| 9 | Does the study provide estimates of the random variability in the data for the main outcomes or exposures (i.e., confidence intervals, standard deviations)? |
| 10 | Does the study provide estimates of the statistical parameters (e.g., regression coefficients)? |
| 11 | Are details of sample size determination included? |
| 12 | Are conclusions substantiated by the data presented in the results? |
| *Study’s Methodological Quality* | |
| 13 | Are study subjects randomized to intervention groups? |
| 14 | Was the procedurę of blinding imposed on subjects/technicians/researchers? |
| 15 | Are the exposure variables reliable/valid? |
| 16 | Are the statistical tests used to assess the main outcomes appropriate? |
| 17 | Are the methods of assessing the outcome variables valid? |
| 18 | Are results adequately compared to previous studies and in relation to theoretical frameworks? |
| *Generalization of results* | |
| 19 | Can the study results be applied to the eligible population? |
| 20 | Can the study results be applied to other relevant populations? |

Appendix 2: Quality assessment items – qualitative study (COREQ)

| No | Item |
| --- | --- |
| *Domain 1: Research team and reflexivity* | |
| *Personal Characteristics* | |
| 1 | Interviewer/facilitator: Which author/s conducted the interview or focus group? |
| 2 | Credentials: What were the researcher’s credentials? |
| 3 | Occupation: What was their occupation at the time of the study? |
| 4 | Gender: Was the researcher male or female? |
| 5 | Experience and training: What experience or training did the researcher have? |
| *Relationship with participants* | |
| 6 | Relationship established: Was a relationship established prior to study commencement? |
| 7 | Participant knowledge of the interviewer: What did the participants know about the researcher? |
| 8 | Interviewer characteristics: What characteristics were reported about the interviewer/facilitator? |
| *Domain 2: study design* | |
| *Theoretical framework* | |
| 9 | Methodological orientation and Theory: What methodological orientation was stated to underpin the study? |
| *Participant selection* | |
| 10 | Sampling: How were participants selected? |
| 11 | Method of approach: How were participants approached? |
| 12 | Sample size: How many participants were in the study? |
| 13 | Non-participation: How many people refused to participate or dropped out? Reasons? |
| *Setting* | |
| 14 | Setting of data collection: Where was the data collected? |
| 15 | Presence of non-participants: Was anyone else present besides the participants and researchers? |
| 16 | Description of sample: What are the important characteristics of the sample? |
| *Data collection* | |
| 17 | Interview guide: Were questions, prompts, guides provided by the authors? Was it pilot tested? |
| 18 | Repeat interviews: Were repeat interviews carried out? If yes, how many? |
| 19 | Audio/visual recording: Did the research use audio or visual recording to collect the data? |
| 20 | Field notes: Were field notes made during and/or after the interview or focus group? |
| 21 | Duration: What was the duration of the interviews or focus group? |
| 22 | Data saturation: Was data saturation discussed? |
| 23 | Transcripts returned: Were transcripts returned to participants for comment and/or correction? |
| *Domain 3: analysis and findings* | |
| *Data analysis* | |
| 24 | Number of data coders: How many data coders coded the data? |
| 25 | Description of the coding tree: Did authors provide a description of the coding tree? |
| 26 | Derivation of themes: Were themes identified in advance or derived from the data? |
| 27 | Software: What software, if applicable, was used to manage the data? |
| 28 | Participant checking: Did participants provide feedback on the findings? |
| *Reporting* | |
| 29 | Quotations presented: Were participant quotations presented to illustrate the themes/findings? Was each  quotation identified? |
| 30 | Data and findings consistency: Was there consistency between the presented data and the findings? |
| 31 | Clarity of major themes: Were major themes clearly presented in the findings? |
| 32 | Clarity of minor themes: Is there a description of diverse cases or discussion of minor themes? |

Appendix 3: Quality assessment scores – quantitative study

| Article | Items | | | | | | | | | | | | | | | | | | | | Total | |
| --- | --- | --- | --- | --- | --- | --- | --- | --- | --- | --- | --- | --- | --- | --- | --- | --- | --- | --- | --- | --- | --- | --- |
|  | 1 | 2 | 3 | 4 | 5 | 6 | 7 | 8 | 9 | 10 | 11 | 12 | 13 | 14 | 15 | 16 | 17 | 18 | 19 | 20 | raw | % |
| Yu-wen Ying, 1999 (1) | 1 | 1 | 1 | 1 | 1 | 1 | 0 | 1 | 1 | 1 | - | 1 | - | - | 0 | 1 | 1 | 0 | - | - | 12 | 75 |
| Ceballo and Hurd, 2008 (3) | 1 | 1 | 1 | 1 | 1 | 1 | 0 | 1 | 0 | 1 | 0 | 1 | - | - | 1 | 1 | 1 | 0 | 0 | 0 | 12 | 66,7 |
| Costigan and Koryzma, 2011 (4) | 1 | 1 | 1 | 1 | 1 | 1 | 0 | 1 | 1 | 1 | 0 | 1 | - | - | 1 | 1 | 1 | 1 | 1 | 1 | 16 | 88,9 |
| Lawton et al, 2016 (5) | 1 | 1 | 1 | 1 | 1 | 1 | 0 | 1 | 1 | 1 | 0 | 1 | - | - | 1 | 1 | 1 | 1 | 1 | 0 | 15 | 83,3 |
| Kiang et al., 2017 (7) | 1 | 1 | 1 | 1 | 1 | 1 | 0 | 1 | 1 | 1 | 0 | 1 | - | - | 1 | 1 | 1 | 1 | 1 | 1 | 16 | 88,9 |
| Stein et al., 2017 (8) | 1 | 1 | 1 | 1 | 1 | 1 | 0 | 1 | 1 | 1 | 1 | 1 | 1 | 1 | 1 | 1 | 1 | 1 | 1 | 1 | 19 | 95 |
| El-Khani et al., 2018 (9) | 1 | 1 | 1 | 1 | 1 | 1 | 1 | 1 | 1 | 1 | 1 | 1 | - | - | 1 | 1 | 1 | 1 | 1 | 1 | 18 | 100 |
| Martinez et al, 2018 (10) | 1 | 1 | 1 | 1 | 1 | 1 | 0 | 1 | 1 | 1 | 1 | 1 | - | - | 1 | 1 | 1 | 1 | 1 | 0 | 16 | 88,9 |
| Malkoff et al., 2019 (11) | 1 | 1 | 1 | 1 | 1 | 1 | 0 | 1 | 1 | 1 | 1 | 1 | - | - | 1 | 1 | 1 | 1 | 1 | 0 | 16 | 88,9 |
| Overall mean | 9 | 9 | 9 | 9 | 9 | 9 | 1 | 9 | 8 | 9 | 4 | 9 | 1 | 1 | 8 | 9 | 9 | 7 | 7 | 4 | 15,6 | 86,2 |

Note: 1 – yes; 0 – no/ unknown; - not applicable.

Appendix 4: Quality assessment scores – qualitative study

| Article | Items | | | | | | | | | | | | | | | | | | | | | | |
| --- | --- | --- | --- | --- | --- | --- | --- | --- | --- | --- | --- | --- | --- | --- | --- | --- | --- | --- | --- | --- | --- | --- | --- |
|  | 1 | 2 | 3 | 4 | 5 | 6 | 7 | 8 | 9 | 10 | 11 | 12 | 13 | 14 | 15 | 16 | 17 | 18 | 19 | 20 | 21 | 22 | 23 |
| Ali, 2008 (2) | 0 | 1 | 0 | 0 | 1 | 0 | 0 | 0 | 0 | 0 | 1 | 0 | 0 | 0 | 0 | 1 | 0 | 0 | 1 | 0 | 0 | 0 | 0 |
| Yakhnich, 2016 (6) | 1 | 1 | 0 | 1 | 1 | 1 | 0 | 1 | 1 | 1 | 1 | 1 | 1 | 1 | 0 | 1 | 1 | 0 | 0 | 1 | 1 | 1 | 0 |
| Overall mean | 1 | 2 | 0 | 1 | 2 | 1 | 0 | 1 | 1 | 1 | 2 | 1 | 1 | 1 | 0 | 2 | 1 | 0 | 1 | 1 | 1 | 1 | 0 |

Note: 1 – yes; 0 – no / unknown; - not applicable.

| Article | Items | | | | | | | | | Total | |
| --- | --- | --- | --- | --- | --- | --- | --- | --- | --- | --- | --- |
|  | 24 | 25 | 26 | 27 | 28 | 29 | 30 | 31 | 32 | raw | % |
| Ali, 2008 (2) | 0 | 0 | 1 | 0 | 0 | 1 | 1 | 1 | 1 | 10 | 31,6 |
| Yakhnich, 2016 (6) | 1 | 1 | 1 | 0 | 0 | 1 | 1 | 1 | 1 | 24 | 75 |
| Overall mean | 1 | 1 | 2 | 0 | 0 | 2 | 2 | 2 | 2 | 17 | 53,3 |

Note: 1 – yes; 0 – no/ unknown; - not applicable.
